# Guidelines for the management of pulmonary nodules detected by low-dose CT lung cancer screening 6th edition: compiled by the Japanese Society of CT Screening

**DOI:** 10.1007/s11604-024-01695-0

**Published:** 2024-12-05

**Authors:** Kazuto Ashizawa, Yuichiro Maruyama, Takeshi Kobayashi, Tetsuro Kondo, Toru Nakagawa, Masayuki Hatakeyama, Masaki Matsusako, Hideyuki Hayashi, Lung Cancer Diagnostic Criteria Subcommittee

**Affiliations:** 1 Department of Clinical Oncology, Nagasaki University Graduate School of Biomedical Sciences, Nagasaki, Japan; 2 Department of Radiology, Asama Nanroku Komoro Medical Center, Komoro, Japan; 3 Department of Diagnostic Radiology, Ishikawa Prefectural Central Hospital, Kanazawa, Japan; 4 Department of Thoracic Oncology, Kanagawa Cancer Center, Yokohama, Japan; 5 Hitachi, Ltd. Hitachi Health Care Center, Hitachi, Japan; 6 Tokyo Anti-Tuberculosis Association, Tokyo, Japan; 7 Department of Radiology, St. Luke’s International Hospital, Chuo, Japan; 8 Department of Radiology, Isahaya General Hospital, Isahaya, Japan; 9 The Japanese Society of CT Screening, Tokyo, Japan

**Keywords:** low-dose CT screening, lung cancer, puimonary nodule, guideline, thin-section CT

## Abstract

**Objective:**

The aim of this special report is to describe the 6th edition of “The Guidelines for the Management of Pulmonary Nodules Detected by Low-Dose CT Lung Cancer Screening “.

**Methods:**

Since the 5th edition six years ago, a review of the literature and consideration of consistency with new evidence led to the revision of the 6th edition.

**Results:**

The main revisions in the 6th edition can be summarized as follows: 1) addition of the section “Recommendations for Low-Dose CT Lung Cancer Screening in Japan”; 2) change in the recommended solid component diameter, and follow-up interval for nodules with a total mean diameter of less than 15 mm and a solid component diameter of less than 8 mm; 3) replacement of the recommended case images; and 4) introduction of the criteria of the Accreditation Council for Lung Cancer CT Screening.

**Conclusion:**

This guideline is gradually gaining acceptance in Japan. This guideline should be applied carefully in clinical practice, considering various factors such as the patient’s condition.

## Introduction

“The Guidelines for the Management of Pulmonary Nodules Detected by Low-Dose CT Lung Cancer Screening “has been updated up to the 5th edition (https://www.jscts.org/pdf/guideline/gls5th201710.pdf). Since the last revision 6 years ago, significant new evidence has emerged worldwide. After reviewing this new evidence and ensuring consistency with current best practices, we have decided to release the 6th edition on April 2024.

The main revisions in the 6th edition are summarized in four key points: (1) the addition of the section “Recommendations for Low-Dose CT Lung Cancer Screening in Japan”; (2) the change in the recommended solid component diameter for a definitive diagnosis in part-solid nodules with a total mean diameter of less than 15 mm from ≥ 5 mm to ≥ 8 mm; (3) the follow-up observation for nodules with a total mean diameter of less than 15 mm and a solid component diameter of less than 8 mm is now recommended at 3, 6, 12, 24, 36, 48, and 60 months; and (4) the replacement of the suggested case images and the introduction of the standards of the Accreditation Council for Lung Cancer CT Screening.

## 1. Recommendations for low-dose CT lung cancer screening in Japan

The effectiveness of low-dose CT lung cancer screening has recently been demonstrated in several large-scale randomized controlled trials in Europe and the USA [1, 2]. According to these results, the Japanese Lung Cancer Society has issued the Lung Cancer Screening Guidelines 2022 [3], with recommendation grades [4] for low-dose CT lung cancer screening published online.

1. Heavy smokers, with a Brinkman index of 600 or more, in the age range of 50–74 are recommended to undergo screening (Recommendation A) owing to evidence of reduced mortality [4]. However, this recommendation is limited to cases where screening is conducted under a sufficient quality control system. If the screening uptake rate is low or follow-up for those requiring detailed examination is insufficient, screening is not recommended. In addition, establishing nationwide criteria for the determination and treatment adaptation is necessary to reduce overdiagnosis. The false-positive rate should be maintained as per the guidelines in the 8th edition of the lung cancer handling rules. Further research is needed to determine the extent of overdiagnosis, false positives, false negatives, incidental findings, and disadvantages such as radiation exposure during screening and follow-up observation in Japan. Moreover, it is necessary to study how to reduce overdiagnosis, lower the false-positive rate, determine appropriate screening intervals, identify target populations (age, smoking history), and assess the feasibility of implementing stratified screening in terms of cost, resources, and quality control on a national level.
2. For nonsmokers, light smokers, and heavy smokers outside the target age group, there is insufficient evidence of mortality reduction; therefore, stratified screening is not recommended (Recommendation I) [4]. Research is needed on the mortality reduction effects for these individuals. In addition, during optional screening, it is necessary to obtain informed consent, similar to the explanation and consent forms outlined in the 8th edition of the lung cancer screening guidelines [5], before proceeding. Furthermore, because the prevalence of lung cancer is lower in non-smokers than in smokers, it is likely that false positive cases will increase, and it is also expected that there will be an increase in cases of overdiagnosis, so it is important to adhere to the diagnosis criteria and treatment indications established by the society. It is important to understand that arbitrarily expanding the scope of diagnosis and treatment will directly lead to an increase in false positives and overdiagnosis, causing harm to patients.

## 2. Guidelines for the management of pulmonary nodules detected by low-dose CT lung cancer screening

Pulmonary nodules are defined as circular or irregularly edged areas of increased attenuation with a maximum diameter of ≤ 3 cm, and spindle-shaped nodules have also been reported [6, 7]. Based on the characteristics of the nodules on thin-section CT (TS-CT) examinations, pulmonary nodules are classified into pure ground-glass nodules (GGNs), part-solid nodules (which include some soft tissue attenuation in addition to ground-glass areas), and solid nodules (which exhibit soft tissue attenuation without ground-glass areas) [8]. The term “ground-glass opacity” in TS-CT refers to a faint area of increased attenuation in which the edges of lung vessels and bronchi are still visible [6]. Pulmonary nodules identified as calcified on TS-CT and considered chronic inflammatory changes, such as those from old pulmonary tuberculosis, are excluded from subsequent follow-up observation.

**Fig. 1:**
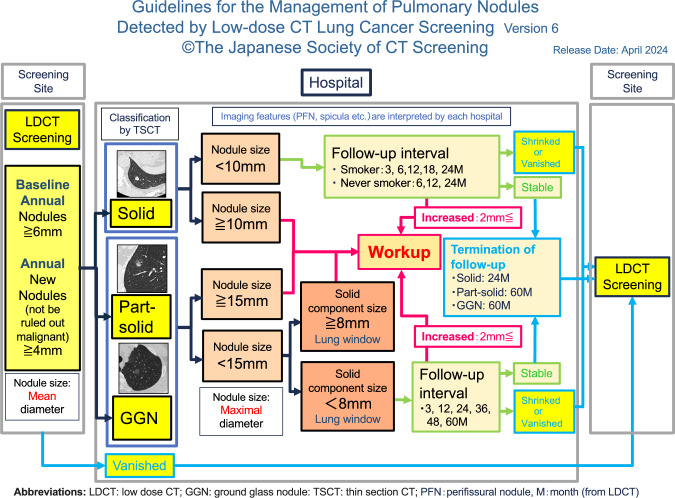
Schema of the 6th edition of the guideline

Schema of the 6th edition of this guideline is shown in Fig. 1

**A. Role of screening institutions (left side in Fig. ** 1**)**.

For pulmonary nodules identified on screening CT images, the criterion for referral to specialized medical institutions for further examination is based on the average of the maximum and minor diameters. If the average size of the pulmonary nodules is less than 6 mm, a follow-up CT examination after 12 months is recommended. However, previous CT images, if any, show no increase even if the nodule measures 6 mm or more, or if it can be determined as an intrapulmonary lymph node, follow-up observation on screening CT is possible, and referral may not be necessary. The adoption of the average of the maximum and minor diameters as size criterion conforms to the statement of the Fleischner Society. [9] Setting the average diameter threshold to 6 mm or more is based on outcome of the International Early Lung Cancer Action Program (I-ELCAP) study, which demonstrated that altering the average diameter from 5 to 6 mm could decrease false positives by 36% without affecting the detection of lung cancers [10]. In addition, the Fleischner Society also utilizes an average diameter of 6 mm [9]. The average size criterion is used exclusively to decide whether to refer patients to a medical institution for detailed examination based on screening CT images. At medical institutions conducting detailed examinations, all size criteria are based solely on the maximum diameter.

For newly appearing nodules measuring 4 mm or more where the possibility of lung cancer cannot be ruled out, patients will be referred to a medical institution if further examination is deemed necessary.

If lung cancer is strongly suspected or if it is determined that urgent examination is required for a patient except for lung cancer, the patient will be referred immediately to a medical institution.

**B. Role of further examination medical institutions (center in Fig.** 1**)**.

At further examination medical institutions, the size criterion is determined by the maximum diameter observed on initial TS-CT scans. Pulmonary nodules with a maximum diameter of 6 mm or more are classified into solid nodules, part-solid nodules, and ground-glass nodules (Fig. 1).

**Fig. 2:**
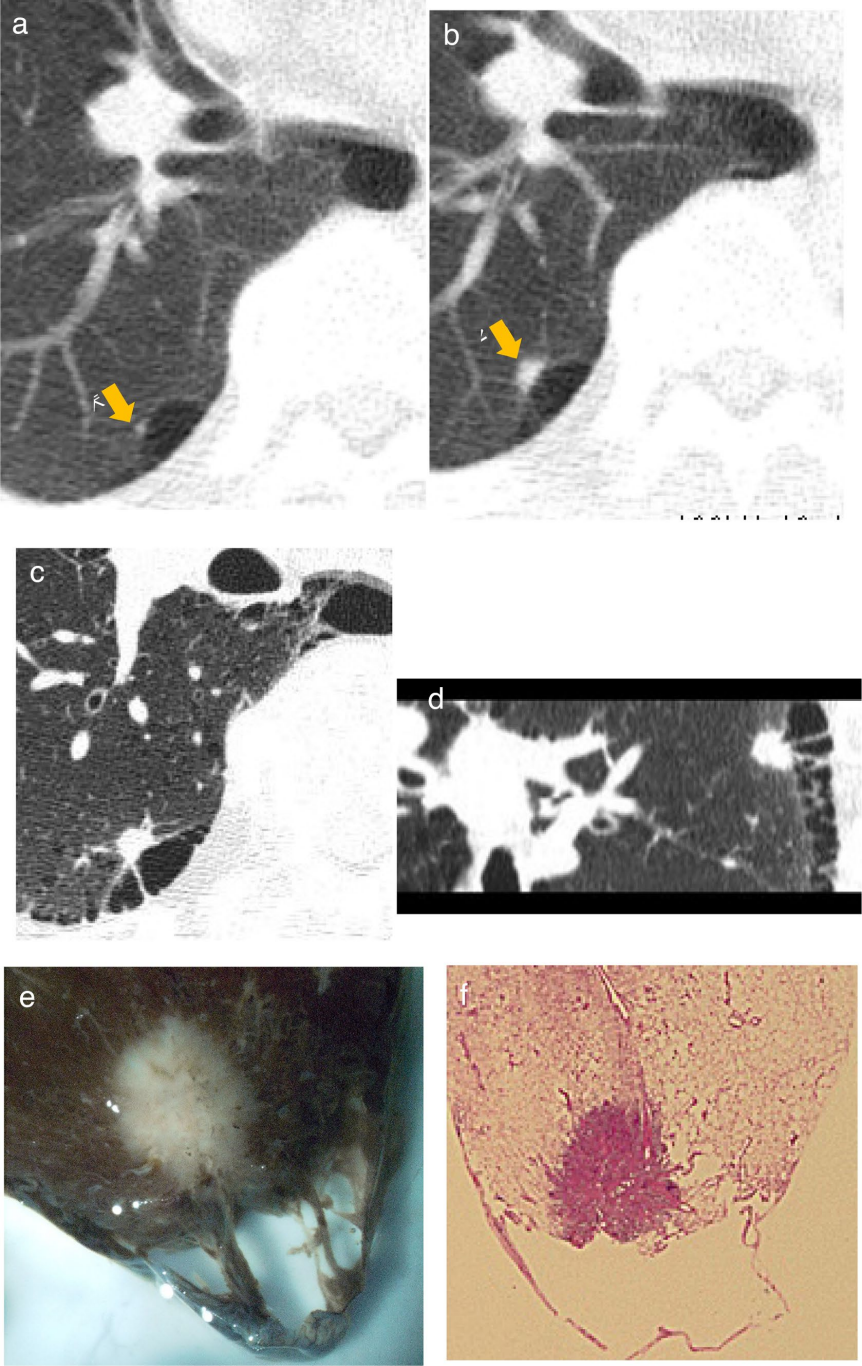
Papillary adenocarcinoma (pT1aN0M0 stage IA1). **a** Low-dose CT screening image (initial screening). **b** Low-dose CT screening image (1 year later). Shooting conditions: 8 mAs (10 mA fixed) CTDIvol = 0.90 mGy, slice thickness: 5 mm. Filtered back projection (FBP) (WW WL) = (1200, −600). The initial low-dose CT screening image (**a**) reveals a small 2.5 mm nodule located in subpleural region of the right lower lobe. One year later, the follow-up low-dose CT screening image (**b**) indicates that the nodule has grown into a 7 mm solid nodule. **c** Thin-section CT (axial image), **d** Thin-section CT (sagittal reconstruction image) slice thickness: 0.625 mm (WW WL) = (1,200, –600). The thin-section CT axial image (**c**) and sagittal reconstruction image (**d**) illustrate a solid nodule measuring 8.6 × 4.3 × 8.5 mm in contact with the bullous wall in subpleural area of the right lower lobe. **e** Extended fixed lung specimen microscopic image, **f** Specimen loupe image, In the extended fixed lung specimen microscopic image (**e**), the solid nodule exhibits slight protrusions on the edges. The specimen loupe image (**f**) illustrates solid growth originating from the bullous wall extending into the lung. The pathological diagnosis was papillary adenocarcinoma

**Fig. 3:**
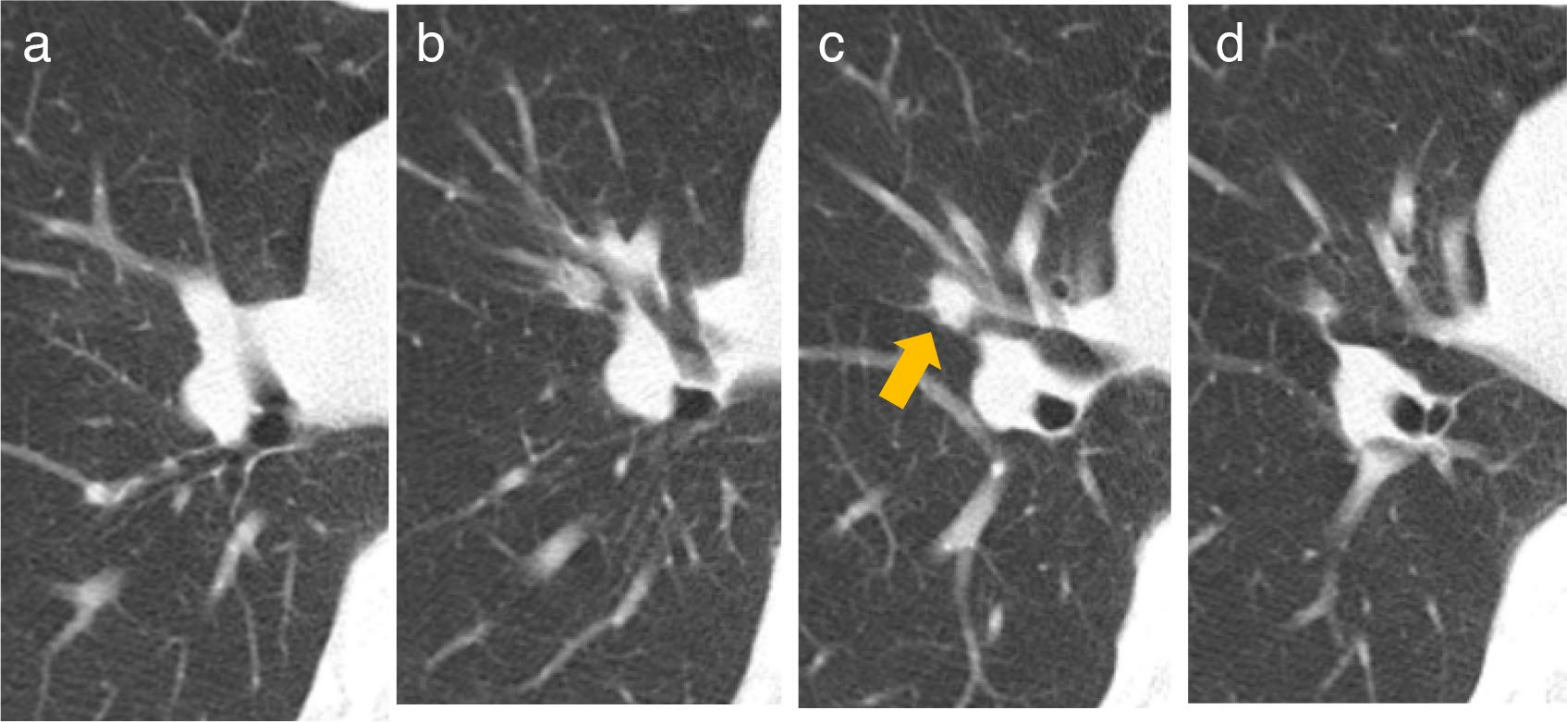
Lung adenocarcinoma (in contact with the vessels at the hilum). **a**–**d** Series of low-dose CT screening images. Shooting conditions: 8 mAs (10 mA fixed) CTDIvol = 0.90 mGy, slice thickness: 5 mm. Filtered back projection (FBP), (WW WL) = (1200–, 600). The sequence of low-dose CT screening images (**a**–**d**) illustrates a 12.7 mm solid nodule located directly beneath the interlobar pleura of the right middle lobe near the hilum. Verifying the vascular course is essential to differentiate it from existing anatomical structures. The pathological diagnosis is lung adenocarcinoma

**Fig. 4:**
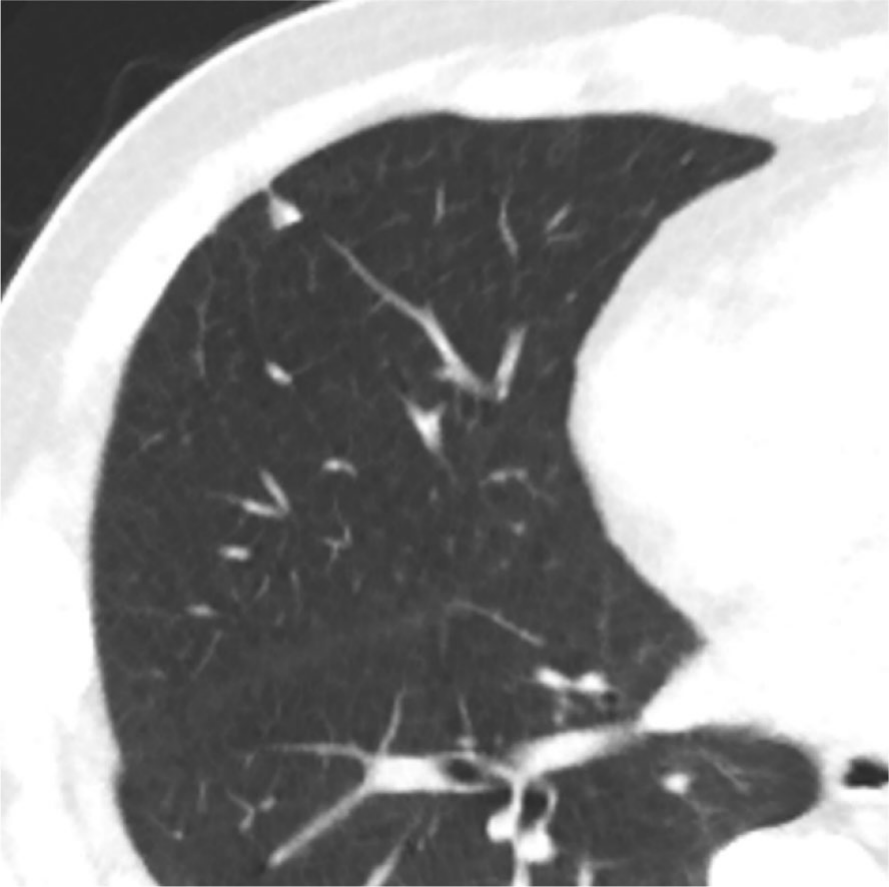
Enlargement of the intrapulmonary lymphatic apparatus (intrapulmonary lymph nodes). Low-dose CT screening image. Shooting conditions: CTDIvol = 0.61 mGy; slice thickness: 2.5 mm; sequential approximation reconstruction (model-based iterative reconstruction: MBIR) (WW WL) = (1,200–, 600). The low-dose CT screening image shows a 6 mm triangular-shaped nodule slightly away from the pleura in the right middle lobe. Linear opacity showing interlobular septa is visible between the nodule and the pleura. The shape strongly suggests an enlargement of the intrapulmonary lymphatic apparatus

**Fig. 5:**
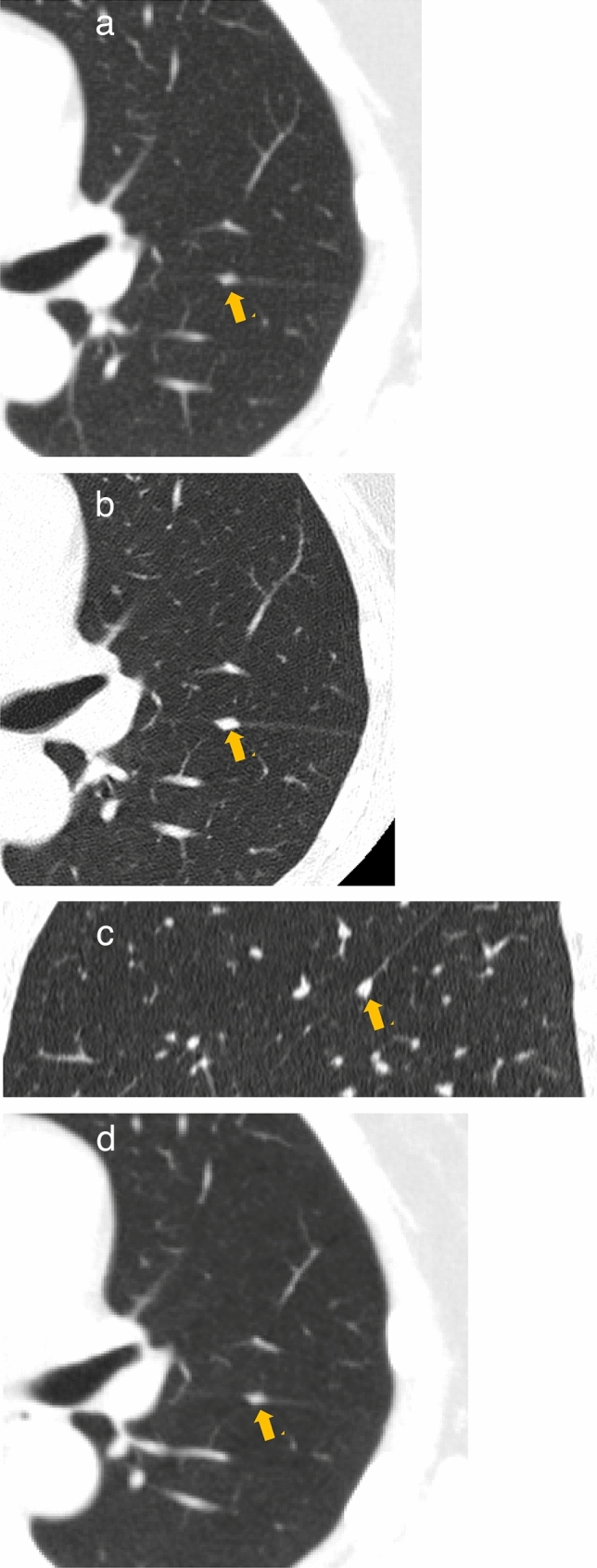
Enlargement of the intrapulmonary lymphatic apparatus (intrapulmonary lymph nodes) (in contact with the pleura). Low-dose CT screening image (**a**) shooting conditions: CTDIvol = 1.2 mGy; slice thickness: 2.5 mm; deep learning reconstruction (DLR) (WW WL) = (1,200–, 600). The low-dose CT screening image (**a**) shows a 6 mm lens-shaped or triangular nodule directly beneath the interlobar pleura in the upper and lower lobes of the left lung. The location and shape strongly suggest an enlargement of the intrapulmonary lymphatic apparatus. **b**, **c** Thin-section CT (axial image, sagittal reconstruction image); slice thickness: 0.625 mm (WW WL) = (1,200–, 600). The thin-section CT axial image (**b**) and sagittal reconstruction image (**c**) illustrate a well-defined polygonal solid nodule measuring 6.3 × 3.1 × 5.1 mm directly beneath the interlobar pleura of the lower left lobe. **d** One year later, the low-dose CT screening image (**d**) shows no change. According to the clinical course, the diagnosis of suspected enlargement of the intrapulmonary lymphatic apparatus was considered

**1) Solid nodules (Figs.** 2–5**)**

On TS-CT, solid nodules with a maximum diameter of 10 mm or more are basically recommended workup for definitive diagnosis. For solid nodules with a maximum diameter between 6 mm and < 10 mm, smokers are recommended to observe over intervals of 3, 6, 12, 18, and 24 months on TS-CT. Meanwhile, nonsmokers are recommended to observe at intervals of 6, 12, and 24 months. In both cases, (i) if an increase of 2 mm or more in the maximum nodule diameter is observed, workup for definitive diagnosis is recommended. (ii) If the nodule remains unchanged over 2 years, follow-up on TS-CT could be terminated, and these cases are recommended to refer back to the screening institution. (iii) If nodule shrinkage or vanishing is observed during follow-up, these cases could be to return to the screening institution for further screening CT evaluation. If pulmonary lymph nodes are strongly suspected according to imaging findings, regardless of size, follow-up on TS-CT is conducted after 3 months. After the confirmation of no change at the 12-month mark, returning to the screening facility is an option (Figs. 4 and 5).

**Fig. 6:**
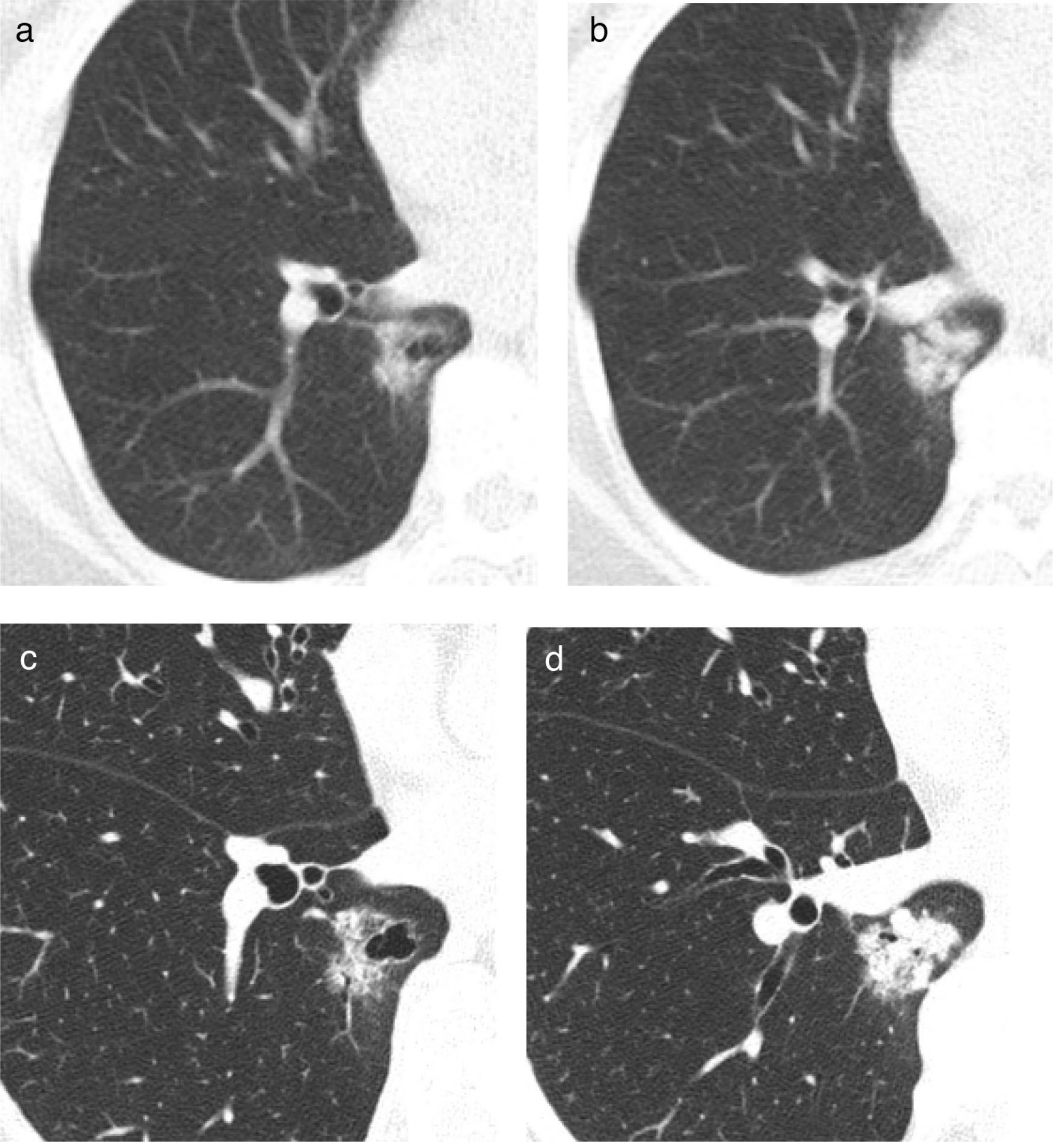
Minimally invasive adenocarcinoma (imaging shows solid component ≥ 8 mm). Low-dose CT screening images (**a**, **b**) Shooting conditions: 8 mAs (10 mA fixed) CTDIvol = 0.90 mGy; slice thickness: 5 mm; filtered back projection (FBP) (WW WL) = (1,200–, 600). The low-dose CT screening images (**a**, **b**) show a 22 × 17 mm nodule in the right lower lobe. The central part of the nodule exhibits a 20 mm solid component, with a faint opacity observed at the periphery. The boundary is distinct, and blood vessel convergence is also visible. **c**, **d** Thin-section CT (axial image); slice thickness: 0.625 mm (WW WL) = (1,200–, 600). The thin-section CT image (**c**) illustrates a part-solid nodule with total dimensions of 25 × 17 × 20 mm, including a solid component measuring 22 mm in the right lower lobe. The edges exhibit ground-glass opacity and bronchial dilatation, and the pleura is invaginated (**d**). Surgical resection was performed. Despite the CT indicating a solid component larger than 8 mm at lung window setting, pathological examination revealed fibrotic foci measuring 2 mm, resulting in a diagnosis of minimally invasive adenocarcinoma (pT1miN0M0)

**Fig. 7:**
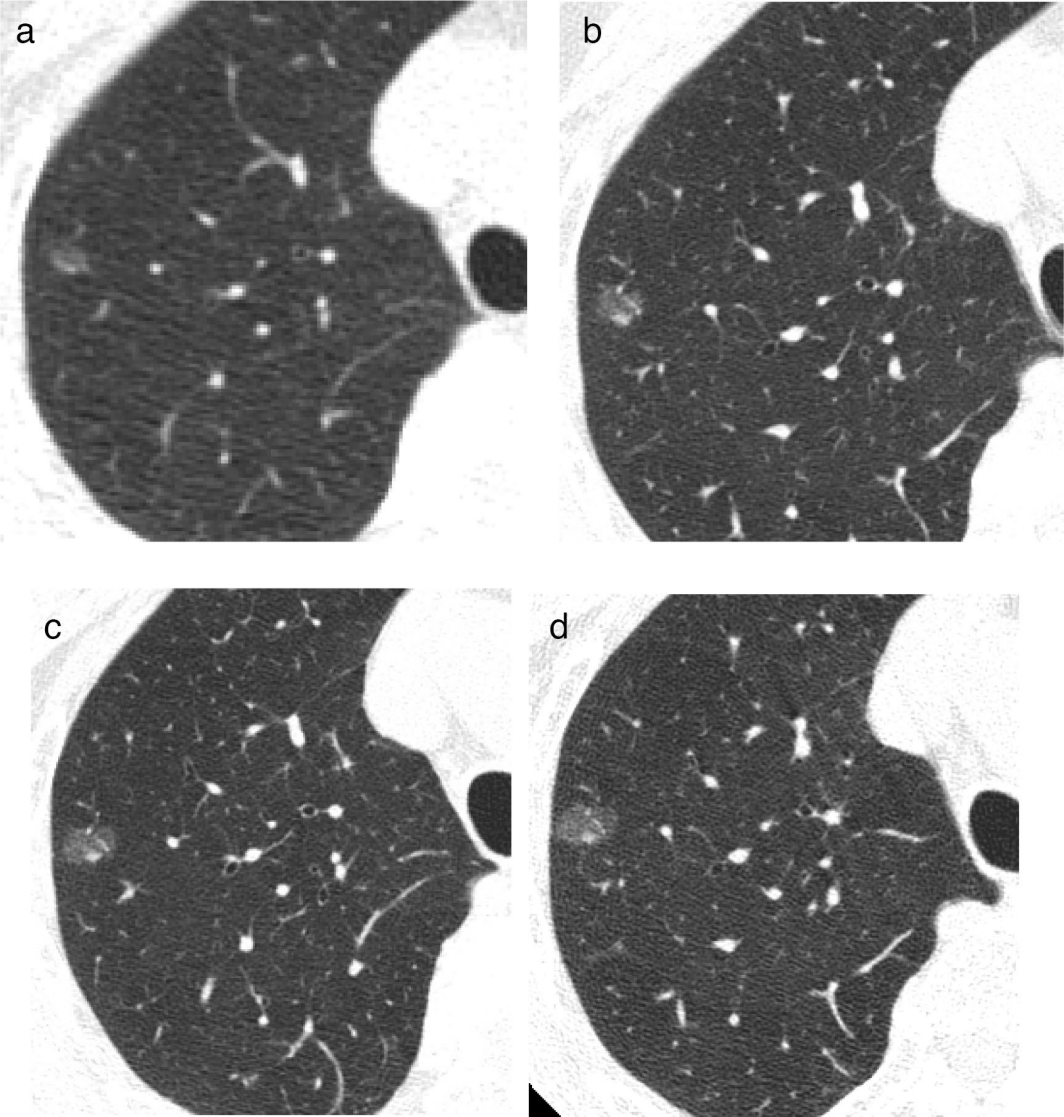
Minimally invasive adenocarcinoma. Low-dose CT screening image (**a**) Shooting conditions: CTDIvol = 0.6 mGy. Slice thickness: 2.5 mm; sequential approximation reconstruction (model-based iterative reconstruction: MBIR) (WW WL) = (1,200–, 600). The low-dose CT screening image (**a**) reveals a faint solitary nodule measuring 7 mm in the right upper lobe. **b**–**d** Thin-section CT (axial image) 1 month after screening (**b**), 2 years later (**c**), and 4 years later (**d**); slice thickness: 0.625 mm (WW WL) = (1,200–, 600). One month after screening, the thin-section CT (**b**) reveals a lesion with ground-glass predominance in the right upper lobe, measuring 8 mm in total diameter with a solid component diameter of 3 mm. Two years later (**c**), the lesion measures 9.5 mm in total diameter, with the solid component diameter remaining at 3 mm. Four years later (**d**), the lesion measures 13 mm in total diameter, with the solid component diameter increasing to 4 mm. Owing to the total diameter increasing by more than 2 mm, surgical resection was performed for definitive diagnosis. Pathological examination revealed fibrotic foci measuring 4 mm, resulting in a diagnosis of minimally invasive adenocarcinoma (pT1miN0M0)

**2) Part-solid nodules (Figs. **6, 7**)**

Part-solid nodules, which are more likely to represent malignant disease [11, 12], necessitate confirmation of any reduction or disappearance on TS-CT after 3 months, because inflammatory lesions are also seen as part-solid nodules on TS-CT. If the nodule with total maximum diameter exceeds 15 mm, a definitive diagnosis is pursued (Fig. 6).

According to the 2015 WHO classification, minimally invasive adenocarcinoma (MIA) and invasive adenocarcinoma (IA) are distinguished by the size of the invasive foci on pathology specimens: 5 mm or less for MIA and more than 5 mm for IA. Solid components observed on TS-CT images may not directly correspond to pathological invasion foci. For nodules with a total maximum diameter of less than 15 mm, if the maximum diameter of the solid component measures 8 mm or more at lung window setting on TS-CT, a definitive diagnosis is conducted. Meanwhile, if the solid component measures less than 8 mm, follow-up observation is recommended. In previous versions, for nonsolid nodules with a total maximum diameter of less than 15 mm, the solid component diameter of 5 mm at lung window setting has been used as the threshold for distinguishing between definitive diagnosis and follow-up observation. However, this revision has adopted 8 mm as the threshold, aligning with Lung-RADS™ [13] and several recent studies suggesting that a solid component diameter of 8 mm at lung window setting is a reasonable cutoff [14, 15].

**Fig. 8:**
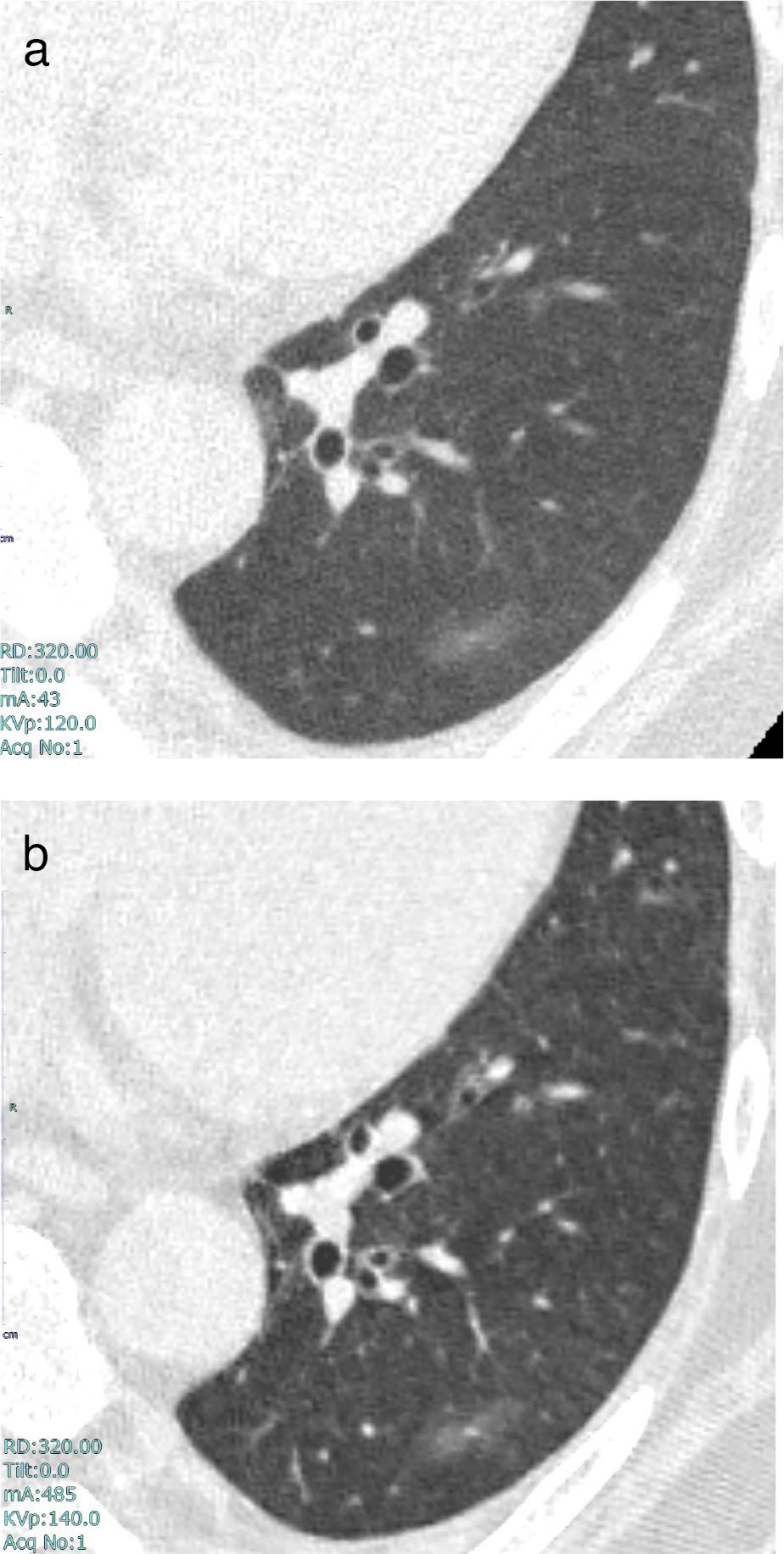
Adenocarcinoma in situ. **a** Low-dose CT screening image (**a**) Shooting conditions: CTDIvol = 2.0 mGy. Slice thickness: 2.5 mm Iterative Reconstruction (ASiR-V) (WW WL) = (1,500–, 600). The low-dose CT screening image (**a**) illustrates a faint pulmonary nodule measuring 18.3 × 9.2 mm in the left lower lobe. The solid component is not visible. **b** Thin-section CT (axial image, regular dose) (**b**); slice thickness: 1.25 mm (WW WL) = (1,500–, 600). The thin-section CT (**b**) reveals a ground-glass nodule measuring 19.6 × 10.7 mm in S10 of the lower left lobe. The central part shows the transparency of pre-existing blood vessels, and no solid component is observed. Owing to the nodule being a ground-glass type with a maximum diameter exceeding 15 mm and demonstrating a trend of growth over time, surgical resection was performed. The pathological diagnosis confirmed adenocarcinoma in situ

**Fig. 9:**
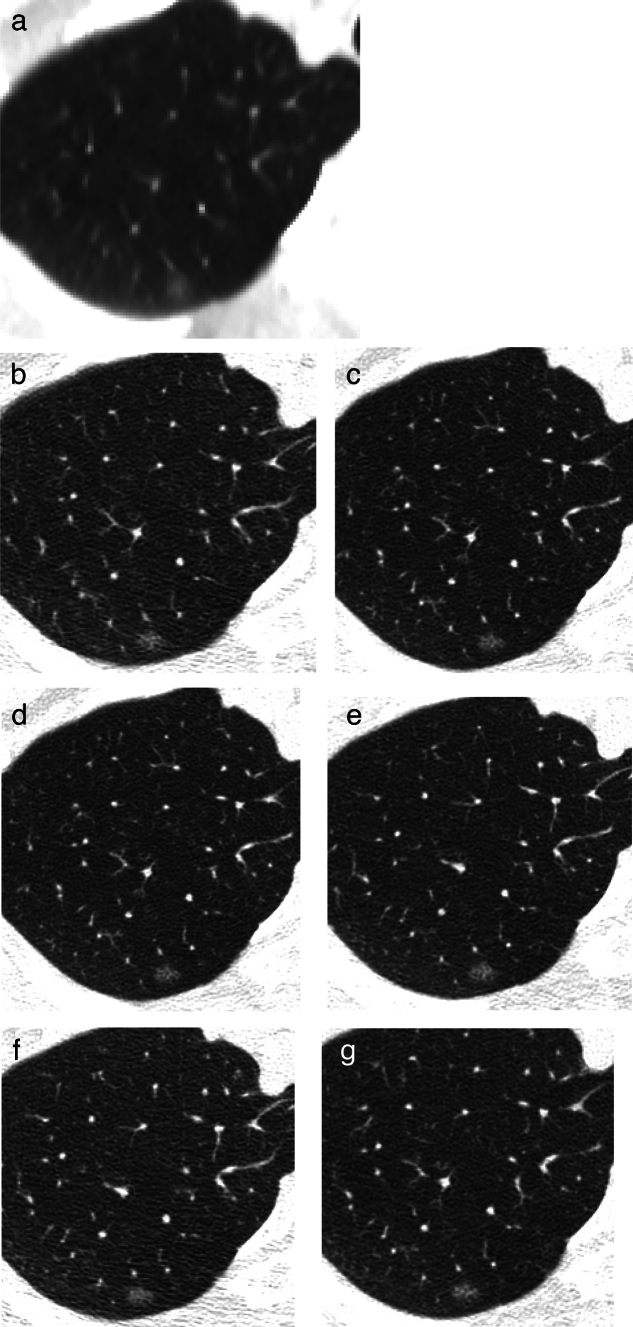
Ground-glass nodule with no temporal change. **a** Low-dose CT screening image (**a**) Shooting conditions: CTDIvol = 0.6 mGy. Slice thickness: 2.5 mm; sequential approximation reconstruction (model-based iterative reconstruction: MBIR) (WW WL) = (1,200–, 600). The low-dose CT screening image (**a**) reveals a faint nodule measuring 6.3 × 5.8 mm in the right upper lobe. **b**–**g** Thin-section CT (axial images) 2 weeks after screening (**b**), 3 months later (**c**), 12 months later (**d**), 24 months later (**e**), 48 months later (**f**), 60 months later (**g**); slice thickness: 0.625 mm (WW WL) = (1,200–, 600). After the detection of an average diameter of ≥ 6 mm in the low-dose CT screening, a thin-section CT was performed 2 weeks later. The CT revealed a ground-glass nodule with total dimensions of 6.2 mm. Subsequent thin-section CT scans conducted at 3, 12, 24, 48, and 60 months showed no changes in the appearance of the nodule

**3) Ground-glass nodules (Figs.** 8, 9**)**

If the maximum diameter of a ground-glass nodule exceeds 15 mm on TS-CT, workup for definitive diagnosis is recommended, even if there is no change or if it increases on TS-CT after 3 months (Fig. 8). If the maximum diameter is less than 15 mm, follow-up observation is conducted at intervals of 3, 12, 24, 36, 48, and 60 months on TS-CT (Case 8): (i) If there is an increase of 2 mm or more or an increase in attenuation, workup for definitive diagnosis is recommended. (ii) If a solid component appears but its maximum diameter is less than 8 mm, further observation could be performed. (iii) For GGNs that remain unchanged over 5 years of follow-up observation, it is recommended that follow-up at medical institutions for further investigations be terminated, but regular low-dose CT screening at the screening institution is recommended.

Although there is few evidence on the long-term follow-up of small pulmonary nodules as detected on CT, it has been reported that of 235 nodules with ground-glass type and part-solid type of 6 mm or more that remained unchanged for 5 years, 5 nodules (approximately 2%) showed growth during follow-up, but no deaths or metastases due to lung cancer were observed [16]. In a study that prospectively followed up 1,229 subsolid nodules in 795 cases for an average of 4.3 years, 56 nodules (5.4%) of 977 homogeneous ground-glass nodules (pure GGN) changed to partial solid type over a period of 3.8 ± 2.0 years, and 16 nodules (19.8%) of 81 heterogeneous ground-glass nodules (heterogeneous GGN) changed to partial solid type over a period of 2.1 ± 2.3 years. Of the 91 nodules that were resected, only 12 were invasive adenocarcinoma (approximately 1% of the total), and all were part-solid nodules [17]. Based on the above reports, we have reconsidered the follow-up period of subsolid nodules beyond 2 years, which has been debated, and have revised the description to suggest follow-up at a precision medical institution at 1-year intervals for up to 5 years.

**4) Definition of enlargement**

The variability between readers in measuring the size of pulmonary nodules was reported as 2.2 mm in one study and 1.72 mm in another [18, 19]. Therefore, an increase of 2 mm or more is considered reasonable for defining total nodule enlargement (Fig. 7). The definition of increase for the solid component follows the criteria used for determining enlargement in solid nodules.

## 3. Points to note in reading, pitfalls

**Fig. 10:**
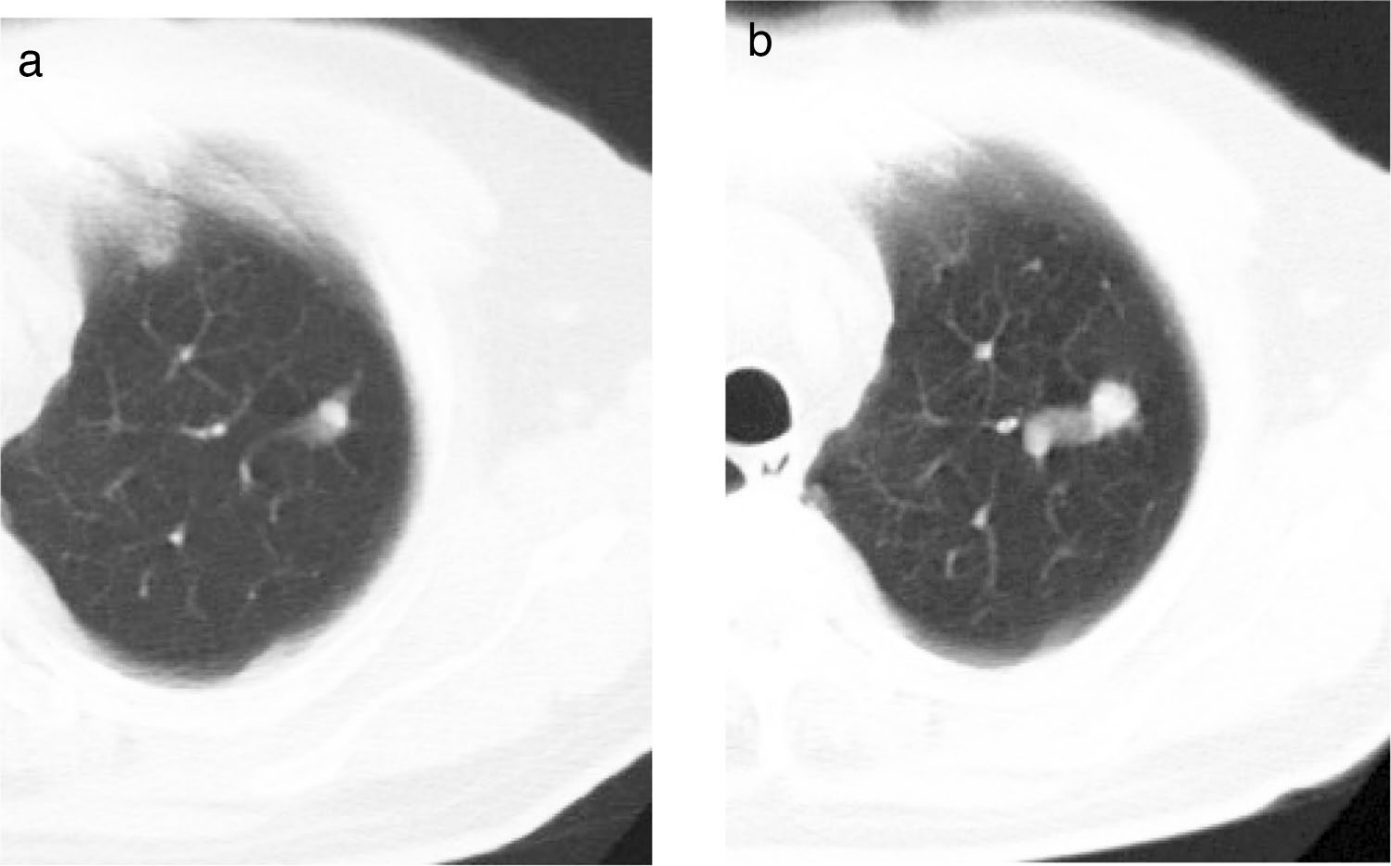
Nodule determined as chronic lesion. **a** Nodule in the left upper lobe was assessed as calcification due to the presence of chronic pulmonary tuberculosis in other areas. This assessment was performed during a CT scan conducted in the previous year, and no comparative reading was performed at that time. **b** One year later, during a follow-up CT scan, the nodule in the left upper lobe was observed to have enlarged. Upon detailed examination, the nodule was diagnosed as small cell lung cancer with bone metastasis, stage IV. Comparative readings are necessary even for nodules classified as “chronic changes,” if previous CT images are available

**Fig. 11:**
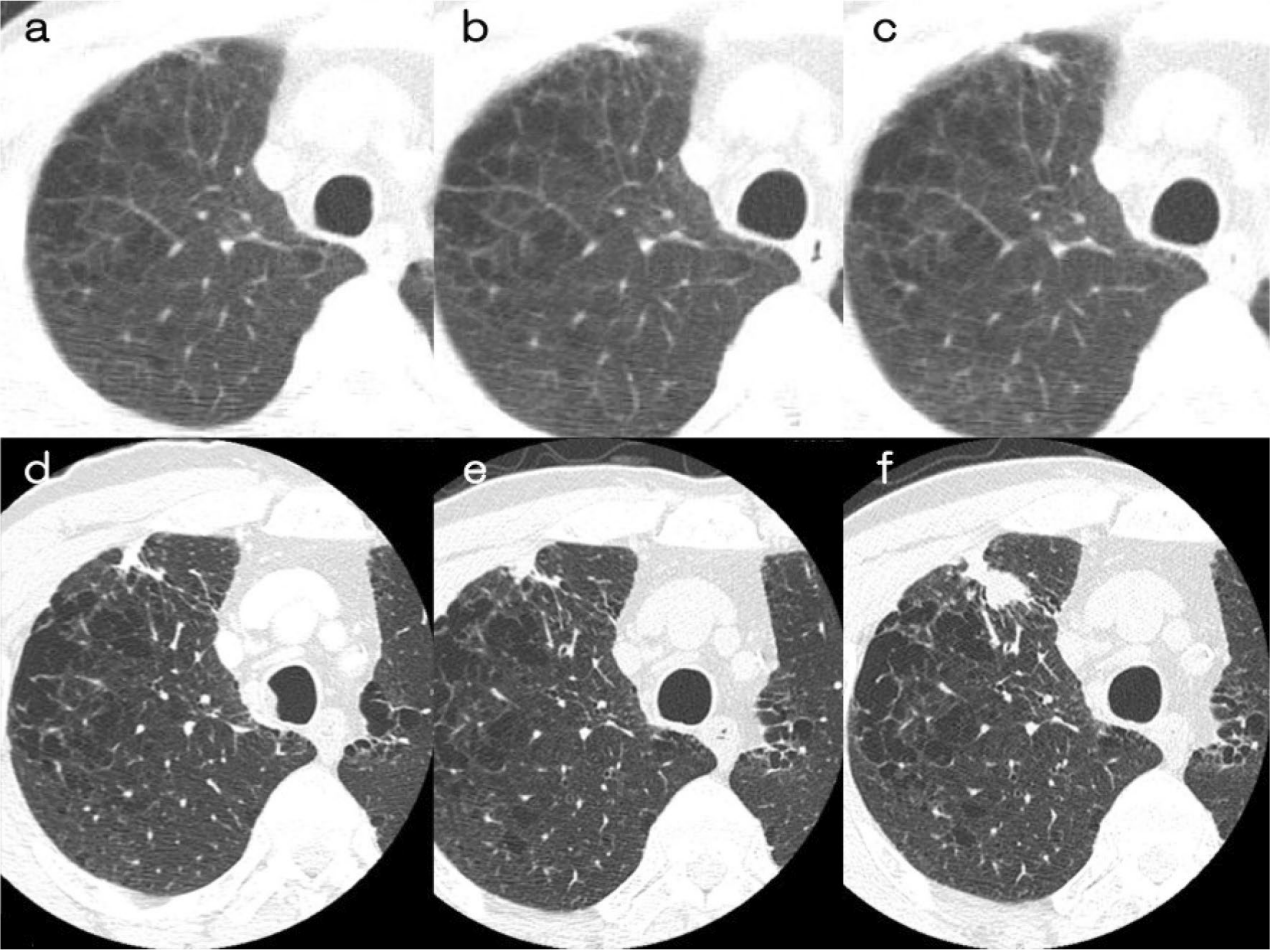
Band opacity appearing in emphysema. **a** A screening CT image taken 2 years prior to referral to a medical institution. Moderate emphysematous changes are observed in the background lung, and a band opacity is noted near the pleura in right S3b. **b** One year before the referral, the screening CT shows an increase in the attenuation value of the band opacity. **c** At the time of referral to the medical institution, the width of the opacity in the dorsoventral direction had increased. **d** a A thin-section CT image taken at the medical institution. **e** Two months later, a follow-up thin-section CT image shows no temporal increase in the band opacity. **f** Six months later, a significant increase in the proximal portion of the band opacity is observed. It was diagnosed as squamous cell carcinoma, cT2aN2M0. It should be noted that lung cancer does not always manifest as a typical pulmonary nodule morphology

**Fig. 12:**
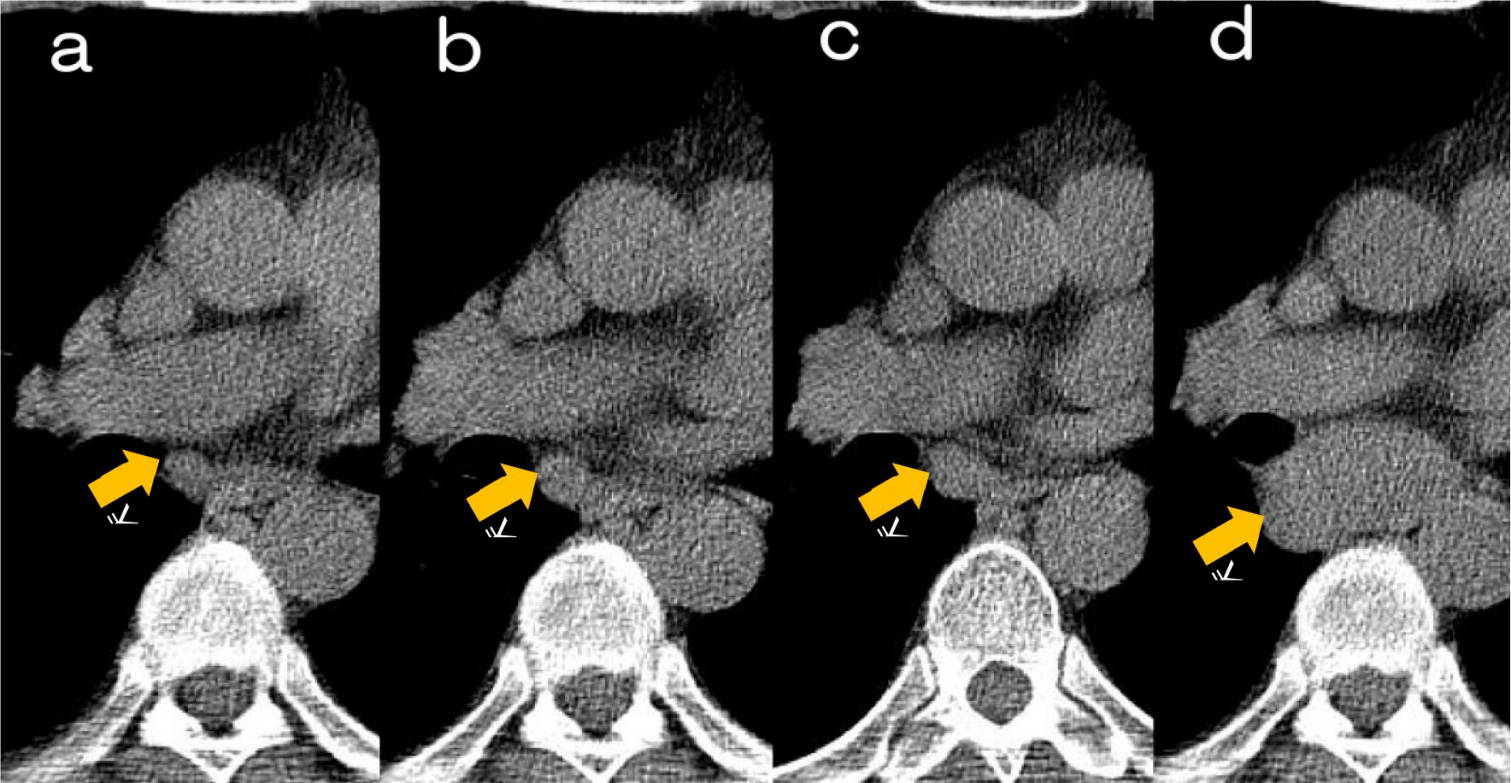
Case with temporal enlargement of mediastinal lymph nodes. **a**–**d** Series of low-dose CT screening images 3 years prior, 2 years prior, 1 year prior, and at the time of diagnosis reveal the progression of findings. **a** A round soft tissue nodule is noted adjacent to the intermediate bronchus (mediastinal lymph node #7) 3 years prior the time of diagnosis. **b**, **c** The round soft tissue nodule showed mild enlargement 2 years prior to the diagnosis (**b**), followed by further enlargement 1 year prior (**c**). **d** At the time of diagnosis, CT shows a large mass with 7 cm in diameter, extending from the tracheal bifurcation to the surrounding vicinity of the opposite main bronchus. Ultrasound-guided bronchoscopic needle biopsy reveals the diagnosis of “mediastinal type” primary lung adenocarcinoma, cT0N3M0. Low-dose CT images should be interpreted with caution, as the image quality of soft tissues such as the mediastinum may degrade, posing challenges for evaluation. Even with low-dose imaging, ensuring image quality sufficient for evaluating soft tissues is desirable

In order to perform highly accurate lung cancer CT screening, we believe it is necessary to clarify the imaging characteristics of lung cancer that is easily overlooked and lung cancer that presents forms other than pulmonary nodules (Figs. 10-12).

The following are points to note when reading CT images.

1. Even if the nodule diameter exceeds 10 mm, it may be overlooked.
2. It is necessary to distinguish between pulmonary blood vessels and pulmonary nodules in areas such as near the hilum.
3. Presence or absence of pulmonary nodules in contact with large blood vessels at the hilum or mediastinum (Fig. 3).
4. Presence or absence of lesions in the trachea or bronchi.
5. Presence or absence of lesions in contact with the pleura (in addition to the peripheral subpleural lesions, interlobar pleura, mediastinum, and diaphragm), but differentiation from intrapulmonary lymph nodes in contact with the peripheral subpleural lesions or interlobar pleura is necessary (Figs. 4, 5).
6. Presence or absence of newly developed localized opacities in existing lesions (inflammatory scars or bullae in the apex of the lung, emphysema, interstitial pneumonia, etc.) (Fig. 11). However, it may progress as a gradual increase in attenuation.
7. Presence or absence of thickening of the walls of the lung cyst (Fig. 2)
8. Consideration of whether it can be judged as an “old lesion” (Fig. 10)
9. Presence or absence of lung nodules in other locations during the follow-up of the lung nodule detected at the initial examination
10. Presence or absence of lung nodules in other locations when one new lung nodule is found

※) Presence or absence of thyroid nodules, calcification of coronary arteries, enlarged adrenal glands, etc.

## Conclusion

“Guidelines for the Management of Pulmonary Nodules Detected by Low-Dose CT Lung Cancer Screening” have been revised for the first time in 6 years. There are many guidelines such as Lung-RADs and those by the Fleischner Society, but in our country, this guideline is gradually gaining traction. This guideline focuses on pulmonary nodules detected by low-dose CT lung cancer screening and should be applied carefully in clinical practice, considering various factors such as the patient’s condition.

## Appendix

### Introduction to facility accreditation standards by the accreditation council for lung cancer CT screening

The Accreditation Council for Lung Cancer CT Screening, a nonprofit organization, accredits radiologists, radiological technologists, and facilities. The accreditation standards for facilities were initially established on April 1, 2018, and revised on June 19, 2019. The accreditation standards for evaluating lung cancer CT screening images are outlined below:

### A. Requirements related to CT equipment and CT imaging

1. Equipment
2. Imaging conditions
3. CT image evaluation

- 1-1 A multi-detector CT (MDCT) with 4 or more detector rows.
- 2-1 Examinations are conducted under conditions where the CTDIvol is 2.5 mGy or less for subjects with a standard body type (BMI 20–22).
- 2-2 Imaging covers from the lung apices to the diaphragmatic part of the lung fields, ensuring no loss of lung field, during a single deep inspiration.
- 2-3 The breath-hold time does not exceed approximately 15 s.
- 2-4 Slice thickness is maintained at 5 mm or less, and slice pitch is reconstructed to be less than the slice thickness.
- 2-5 Annually, following accreditation, the institution must report the dose indicators (CTDIvol, DLP) for 20 consecutive subjects examined during a specified period to the organization, using a format defined by the organization.
- 3-1 Submission of DICOM data of CT images from two male subjects (standard body type and BMI above 25), including dose indicators, for review by the organization.
- 3-2 In addition, within 2-year post-accreditation, submission of DICOM data of standard chest phantom CT images along with dose indicators is required.

### B. Requirements for CT screening implementers

1. Responsibility
2. Physicians
3. Technologists
4. Evaluators

- 1-1 The person responsible for CT screening ensures continuous monitoring and guidance to maintain proper imaging, reading, and quality control standards.
- 2-1 At least one accredited physician is employed, either on a full-time or part-time basis.
- 3-1 At least one accredited technologist is employed on a full-time basis.
- 4-1 Dual readings are conducted, with at least one of the readers being an accredited physician.

### C. Quality control requirements for CT screening

1. Statistical data
2. Review meetings

- 1-1 Annual statistical data are compiled and reported.
- 1-2 Each year, the organization mandates reporting of the year’s data in a specified format.
- 2-1 At least one facility review meeting is held annually to reevaluate diagnosed lung cancer cases, and records of the meeting are kept.
- 2-2 Each year, the organization requires the submission of the annual records in a specified format.

### D. Requirements for CT screening performance

At least 50 CT screenings are performed annually.

### E. Safety management requirements for CT screening

1. Quality control of medical devices

2. Management system of the screening organization

- 1-1 Regular inspections and periodic quality control of the equipment are conducted and documented.
- 2-1 The management system of the screening organization is well-established.
- 2-2 The facility has a system in place to compile and regularly report CT screening statistical data.

For more details, please visit the following website: Facility Accreditation Application (the Accreditation Council for Lung Cancer CT Screening) [ct-kensin-nintei.jp].
